# Intrusive Images of a Spider and an Octopus: A Unique Presentation of Obsessive-Compulsive Disorder (OCD)

**DOI:** 10.7759/cureus.81673

**Published:** 2025-04-03

**Authors:** Avijoy Roy Choudhury

**Affiliations:** 1 Psychiatry, Graylands Hospital, Perth, AUS

**Keywords:** cognitive behavioural therapy (cbt), intrusive imagery, ocd/ anxiety disorders, psychiatry & mental health, selective serotonin reuptake inhibitor (ssri), visual and auditory hallucinations

## Abstract

Obsessive-compulsive disorder (OCD) is a severe and disabling psychiatric disorder that impacts people all around the world. OCD has several different phenotypes, with intrusive visual images being a presentation of the disease. In this paper, a case is presented of a patient who suffers from intrusive visual images of a large spider and octopus with visualization of the tentacle on her body. These visual images were identified as a symptom of OCD; therefore, this paper aims to present this unique presentation of the disease.

## Introduction

Obsessive-compulsive disorder (OCD) is a severe and disabling psychiatric disorder that is characterized by obsessions and compulsions that are impairing, distressing, time-consuming, and ego-dystonic [1]. Obsessions are thoughts, urges, or images of a persistent, intrusive, and recurrent nature. Compulsions are behaviors used to manage obsessions and include mental acts or ritualized behaviors. OCD impacts approximately 3% of the global population and can have a hugely impactful effect on the quality and quantity of life, especially if untreated [2]. That being said, OCD remains underdiagnosed and undertreated globally. This is due to the diverse presentation of the disorder and the stigma surrounding the condition [3]. The typical onset of OCD is in childhood, adolescence, or early adulthood. The median onset of OCD is 19 years [4].

OCD can present in various forms, but the core features of obsessions and compulsions are always present. The common phenotypes of OCD are contamination OCD, symmetry and order OCD, harm OCD, and checking OCD. Contamination OCD is where patients have worries over germs and bacteria. Symmetry and order OCD involves patients having obsessions over exactness leading to a need for symmetry and a particular order of things. Harm OCD occurs with patients have intrusive thoughts about causing harm to themselves or others. Checking OCD is characterized by repetitive obsessions resulting in compulsive checking and rechecking to ensure things are correct [5].

Numerous scales are available to evaluate the severity of OCD, with the Yale-Brown Obsessive-Compulsive Scale being the most widely used. This scale evaluates the severity of disease by looking at frequency, intensity, and impact on life [6]. 

Treatment around OCD is multifactorial, with cognitive behavioral therapy (CBT) focusing on exposure and response prevention as a major component. Psychopharmacology, such as selective serotonin reuptake inhibitors (SSRIs) and tricyclic antidepressants are also commonly utilized in the treatment of OCD [7].

This case report outlines a patient with an unusual presentation of OCD. The aim of this case report is to record an uncommon presentation, raise understanding around OCD, and highlight the impact of OCD.

## Case presentation

A 49-year-old female with no previous mental health diagnosis was brought into a psychiatric hospital for assessment. The patient reported a year-long history of experiencing visual images in the form of a large spider and octopus with the visualization of tentacles on her body. She denied feeling any sensation over her body. The patient reported that these visual images were reoccurring and intrusive in nature and occurred multiple times in an hour. The patient felt that the frequency of the visual hallucinations was worsening and was impacting her day-to-day activities. She reported that the visual images were associated with her mood state and worsened with increased anxiety and low mood.

She reported low mood, low energy levels, suicidal ideations, and poor sleep secondary to the visual images. The patient denied any auditory hallucinations, paranoia, thought insertion, or thought removal. There was no evidence of disordered thought, paranoia, or disordered speech.

The patient reported no history of drug or alcohol use. A negative urine drug screen was produced. The patient reported that she had undertaken ayahuasca 3 years prior to the beginning of symptoms. She also reported smoking five to six cigarettes per day.

An EEG was performed which was negative for any seizure activity. An MRI Brain was also conducted but no abnormalities were found. There were no significant findings in blood labs or during physical examination. The patient's past medical history included admission to a general hospital for the treatment of urosepsis.

On mental state examination at admission, the patient appeared as a middle-aged Caucasian female. She was well-groomed, casually dressed, and had blonde hair. The patient was pleasant, cooperative, and easy to engage, with good eye contact. There were no overt psychotic symptoms elicited. Her speech had a normal rate, rhythm, tone, and volume, and was spontaneous. She reported her mood as “okay” and had an affect that was somewhat anxious but overall was euthymic and reactive. She was reporting visual images of a spider and an octopus. She was not observed to be responding to unseen stimuli during the assessment. Her thought form was linear and logical. There was no paranoid or delusional thought content. She was not expressing active suicidal ideations or self-harm thoughts. She was orientated to time, place, and person. The patient had insight and her judgment was intact.

The patient was previously trialed on numerous antipsychotic medications, including olanzapine, risperidone, and quetiapine, at various therapeutic doses with no effect. As a part of the patient's management during this presentation, the patient was started on clomipramine 75 mg twice daily by the treating team. Following this modality of treatment, there was a reported 60% reduction in the patient's symptoms, including the visual images.

Given the uncommon nature of the presentation, establishing diagnostic clarification was challenging. Extensive examination and investigations were performed to rule out organic causes such as infection or epilepsy. Examinations completed included a full physical neurological examination, cranial nerve examination, cardiovascular examination, respiratory examination, and abdominal examination. The diagnosis of the symptoms being of a psychotic nature was unlikely due to the lack of response from numerous sufficient trials of antipsychotic medications. Furthermore, there was no evidence of thought disorder. The patient also had good insight. A diagnosis of OCD was established given that the visual images were repetitive, intrusive, and ego-dystonic in nature. Further backing of the OCD diagnosis was provided by the effectiveness of treatment from clomipramine, which is one of the main medications used in the treatment of OCD.

## Discussion

Intrusive visual images are a common symptom of OCD. These images can be vivid and distressing. Often, the images are violent or sexual in nature. The images are ego-dystonic as there is an inconsistency between the images and the person's actual desires or values. Visual images caused by OCD can be significant and can result in impactful psychological problems that can have a hugely negative result on the quality of life of the individual [8].

Differentiating between visual images caused by OCD and those caused by psychosis is important and often challenging. This diagnostic clarification is essential as it is hugely impactful in the management and treatment of the disease.

A major differentiating factor is the nature of the experience. In OCD, the images are intrusive, unwanted, and distressing, and the patient is aware they are not real. In contrast, in psychosis, the images present as visual hallucinations that are often perceived as real. Insight is a major differentiating factor between OCD and psychosis, with people suffering from OCD generally having a much higher level of insight [9].

The duration and frequency of the visual images are also important in establishing diagnostic clarification. Intrusive images related to OCD are often episodic in nature and may be tied to a specific trigger. They also may be tied to a compulsion that is often used to reduce the anxiety caused by the images. In comparison, images caused by psychosis tend to be persistent in nature and happen frequently. They may persist for hours or days until treated. Images related to psychosis are often difficult to suppress without medication [9]. 

The associated symptoms are also major diagnostic factors when differentiating between visual images caused by OCD versus psychosis. This is particularly relevant in looking at visual images caused by psychosis as associated symptoms include disorganized thinking, delusions, paranoia, confusion, or disorganized speech. These symptoms are not usually associated with OCD [10].

Finally, response to treatment is also something that is hugely significant in differentiating between psychosis and OCD. The treatment of OCD includes the use of CBT, particularly exposure and response prevention therapy. Exposure and response prevention therapy involves gradually exposing individuals with OCD to obsession-provoking situations while preventing the compulsion. This process gradually reduces anxiety while aiding in the development of coping strategies for individuals [11]. Studies suggest that CBT is effective for approximately 60-70% of people with OCD [12]. 

In addition to CBT, OCD is also often treated using antidepressants such as SSRIs (like escitalopram, sertraline, and fluvoxamine) and tricyclic antidepressants, especially clomipramine [13]. If symptoms are of a psychotic nature, then these modes of treatment will be unlikely to be effective and often require antipsychotic medication [14].

## Conclusions

In this paper, we have reported the case of a 49-year-old female who presented with intrusive visual images caused by OCD. Diagnostic clarification was the major challenge in this case, and the diagnosis was hugely determined by response to treatment, insight, and the intrusive nature of the images. Treatment for OCD and psychotic disorders are significantly different; therefore, establishing diagnostic clarification is essential to ensure that patients are treated effectively. Given the challenges in diagnostic clarification, reporting this case is important for developing further understanding related to OCD presentations.

## References

[1] Jalal B, Chamberlain SR, Sahakian BJ (2023). Obsessive-compulsive disorder: etiology, neuropathology, and cognitive dysfunction. Brain Behav.

[2] Hirschtritt ME, Bloch MH, Mathews CA (2017). Obsessive-compulsive disorder: advances in diagnosis and treatment. JAMA.

[3] Mathews C (2021). Obsessive-compulsive disorders. Continuum (Minneap Minn).

[4] Ruscio AM, Stein DJ, Chiu WT, Kessler RC (2010). The epidemiology of obsessive-compulsive disorder in the National Comorbidity Survey Replication. Mol Psychiatry.

[5] Abramowitz JS (2006). The psychological treatment of obsessive-compulsive disorder. Can J Psychiatry.

[6] Koran LM, Thienemann ML, Davenport R (1996). Quality of life for patients with obsessive-compulsive disorder. Am J Psychiatry.

[7] Fatori D, Costa DL, Asbahr FR (2020). Is it time to change the gold standard of obsessive-compulsive disorder severity assessment? Factor structure of the Yale-Brown Obsessive-Compulsive Scale. Aust N Z J Psychiatry.

[8] Rachman S (2007). Unwanted intrusive images in obsessive compulsive disorders. J Behav Ther Exp Psychiatry.

[9] Lipton MG, Brewin CR, Linke S, Halperin J (2010). Distinguishing features of intrusive images in obsessive-compulsive disorder. J Anxiety Disord.

[10] Arciniegas DB (2015). Psychosis. Continuum (Minneap Minn).

[11] Abramowitz JS, Franklin ME, Schwartz SA, Furr JM (2003). Symptom presentation and outcome of cognitive-behavioral therapy for obsessive-compulsive disorder. J Consult Clin Psychol.

[12] Keeley ML, Storch EA, Merlo LJ, Geffken GR (2008). Clinical predictors of response to cognitive-behavioral therapy for obsessive-compulsive disorder. Clin Psychol Rev.

[13] Del Casale A, Sorice S, Padovano A (2019). Psychopharmacological treatment of obsessive-compulsive disorder (OCD). Curr Neuropharmacol.

[14] Jukic M, Milosavljević F, Molden E, Ingelman-Sundberg M (2022). Pharmacogenomics in treatment of depression and psychosis: an update. Trends Pharmacol Sci.

